# The association between COVID-19 vaccine/infection and new-onset asthma in children - based on the global TriNetX database

**DOI:** 10.1007/s15010-024-02329-3

**Published:** 2024-06-21

**Authors:** Chiao-Yu Yang, Yu-Hsiang Shih, Chia-Chi Lung

**Affiliations:** 1 Department of Occupational Health Nursing Center, Institute of Public Health, Chung Shan Medical University Hospital, Taichung City, Taiwan; 2 Department of Public Health, Chung Shan Medical University, No. 110, Sec. 1 Jianguo N.Rd., Taichung City, 40201 Taiwan; 3 Department of Obstetrics and Gynecology, Taichung Veterans General Hospital, Taichung City, Taiwan; 4 Department of Health Policy and Management, Chung Shan Medical University, Taichung City, Taiwan; 5 Department of Family and Community Medicine, Chung Shan Medical University Hospital, Taichung City, Taiwan

**Keywords:** SARS-CoV-2, COVID-19, COVID-19 vaccine, New-onset asthma, Asthma, Children

## Abstract

**Introduction:**

The COVID-19 pandemic has underscored the importance of its potential long-term health effects, including its link to new-onset asthma in children. Asthma significantly impacts children’s health, causing adverse outcomes and increased absenteeism. Emerging evidence suggests a potential association between COVID-19 infection and higher rates of new-onset asthma in adults, raising concerns about its impact on children’s respiratory health.

**Methods:**

A retrospective cohort study design was employed, using electronic medical records from the TriNetX database, covering January 1, 2021, to December 31, 2022. Two cohorts of children aged 5 to 18 who underwent SARS-CoV-2 RT-PCR testing were analyzed: unvaccinated children with and without COVID-19 infection, and vaccinated children with and without infection. Propensity score matching was used to mitigate selection bias, and hazard ratio (HR) and 95% CI were calculated to assess the risk of new-onset asthma.

**Results:**

Our study found a significantly higher incidence of new-onset asthma in COVID-19 infected children compared to uninfected children, regardless of vaccination status. In Cohort 1, 4.7% of COVID-19 infected children without vaccination developed new-onset asthma, versus 2.0% in their non-COVID-19 counterparts within a year (HR = 2.26; 95% CI = 2.158–2.367). For Cohort 2, COVID-19 infected children with vaccination showed an 8.3% incidence of new-onset asthma, higher than the 3.1% in those not infected (HR = 2.745; 95% CI = 2.521–2.99). Subgroup analyses further identified higher risks in males, children aged 5–12 years, and Black or African American children. Sensitivity analyses confirmed the reliability of these findings.

**Conclusion:**

The study highlights a strong link between COVID-19 infection and an increased risk of new-onset asthma in children, which is even more marked in those vaccinated. This emphasizes the critical need for ongoing monitoring and customized healthcare strategies to mitigate the long-term respiratory impacts of COVID-19 in children, advocating for thorough strategies to manage and prevent asthma amidst the pandemic.

**Supplementary Information:**

The online version contains supplementary material available at 10.1007/s15010-024-02329-3.

## Introduction

The COVID-19 pandemic continues to pose significant challenges to global health. SARS-CoV-2, primarily recognized as a respiratory pathogen, is also associated with a spectrum of cardiovascular, pulmonary, neurologic, and metabolic complications [1]. 

Asthma, marked by airway inflammation and hyperreactivity, profoundly affects children’s health across the globe. Childhood asthma significantly impacts long-term health, leading to deteriorations in health status, increased obesity rates, and a 40–50% rise in absenteeism in early adulthood [2]. These outcomes highlight the profound influence of early health conditions on future health and societal outcomes. Historical data indicate a link between respiratory viruses, including rhinovirus and respiratory syncytial virus, and the exacerbation of asthma symptoms as well as the onset of asthma [3, 4]. Mechanisms involved may include epithelial inflammatory responses, leukocyte activation leading to cytokine and inflammatory factor production, and proliferation of fibroblasts and smooth muscle cells contributing to airway tissue remodeling [3]. 

Although the processes underlying the increase in asthma incidence from COVID-19 infection remain unclear and underexplored, they could be similar to those observed with other viral infections. Emerging evidences indicate a potential link between COVID-19 infection and an elevated rate of new-onset asthma in adults, possibly resulting from the virus’s inflammatory response, which may induce permanent alterations in airway functionality and sensitivity [5–7]. To date, there is limited and inconclusive literature on the risk of asthma following COVID-19 infection in children. A retrospective cohort study found that SARS-CoV-2 PCR positivity was not associated with a new asthma diagnosis in children aged 1-16 [8]. But another study indicated that an asthma diagnosis was negatively associated with COVID-19-related hospitalization in children [9]. 

COVID-19 vaccines are widely administered and have significantly reduced the risk of severe illness, hospitalization, and mortality [10], literature indicates that a minority of individuals may develop adverse effects after vaccination, such as myocarditis [11], and symptoms related to the menstrual cycle (irregular bleeding, dysmenorrhea, mood changes) [12]. However, the long-term effects of the COVID-19 vaccine remain to be further observed. A meta-analysis found that vaccination against COVID-19 consistently reduced the risk of long COVID symptoms in adult [13]. Despite promising results, further research is needed to fully understand the vaccine’s effectiveness in preventing long COVID across different populations and to identify any potential long-term side effects, especially in children.

We hypothesized that the risk of new-onset asthma in children increases following COVID-19 infection due to virus-induced inflammatory responses. Therefore, we performed a large, retrospective cohort study to assess the relationship between COVID-19 infection and the incidence of new-onset asthma in children. Additionally, we examined whether COVID-19 vaccination could mitigate this risk, aiming to provide a comprehensive understanding of the potential protective effects of vaccination against respiratory complications in this population.

## Methods

### Data source

This study utilized data from the TriNetX databasehttps://trinetx.com/?mc_cid=7e2ecd5bc5&mc_eid=%5BUNIQID%5D, a global health research network providing access to de-identified electronic medical records. This extensive database covers approximately 250 million individuals across 120 healthcare organizations worldwide, making it the largest COVID-19 dataset available. The available data include demographics, diagnoses (utilizing ICD-10-CM codes), procedures (using ICD-10-PCS or CPT codes), medications (coded in the Veterans Affairs National Formulary), laboratory measurements (LOINC-coded), and healthcare utilization details. The children in the study were recruited from the TriNetX US Collaborative Network, a real-time, multicenter national health network encompassing data from 64 healthcare organizations across all 50 states of the United States.

Data analysis was conducted in February 2024, and covering the study period from January 1, 2021, to December 31, 2022. Patients or the public WERE NOT involved in the design, or conduct, or reporting, or dissemination plans of our research.

### Study design

**Fig. 1:**
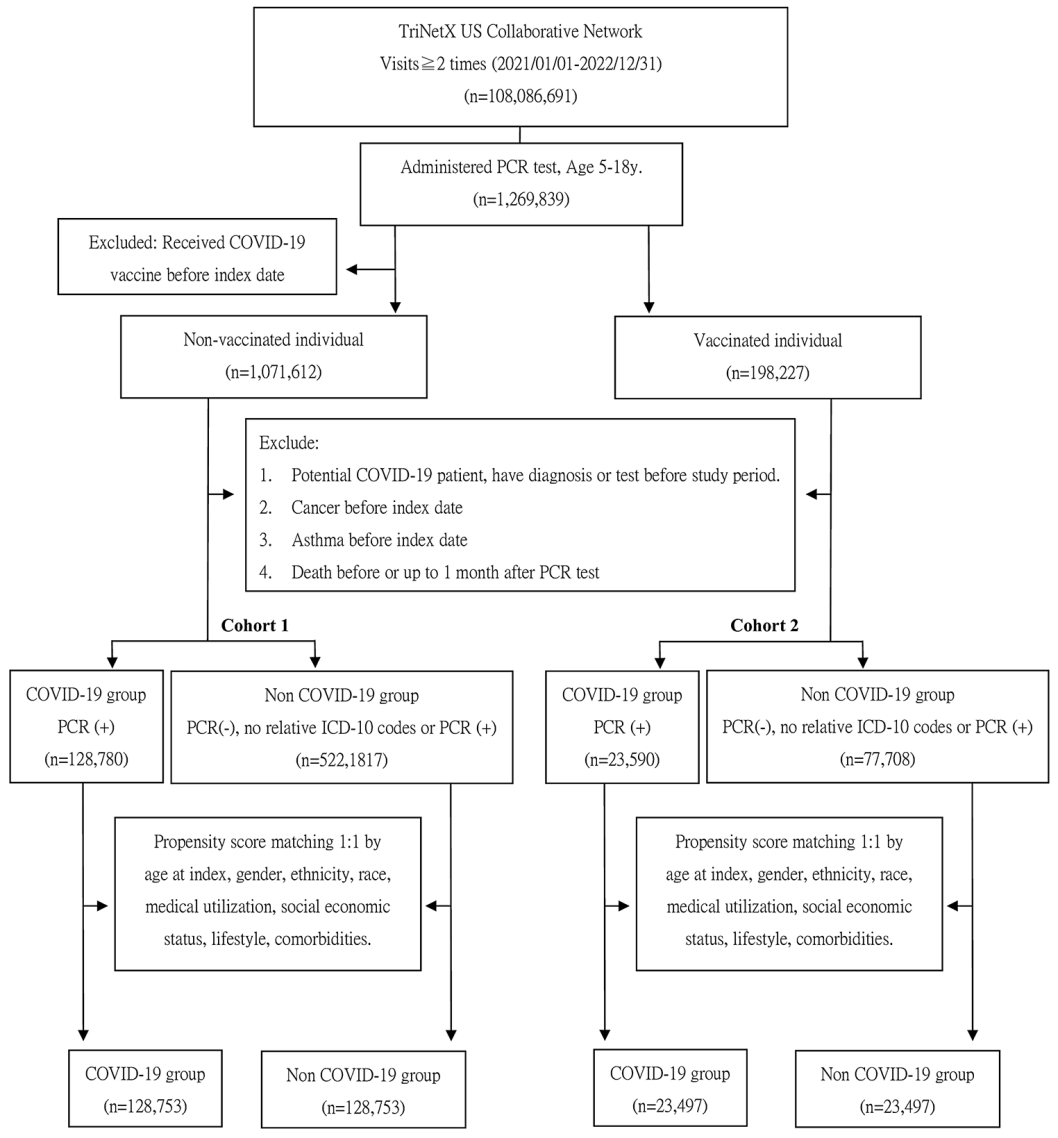
Study Flowchart

This retrospective cohort study employed data from the TriNetX database, a comprehensive global health research network offering access to electronic medical records from various healthcare organizations. It aimed to evaluate the association between COVID-19 vaccination/infection and the incidence of new-onset asthma in children. Figure 1 presents the flow chart detailing the process of our cohorts’ construction. The study constructed two cohorts comprising children aged 5 to 18 who underwent SARS-CoV-2 RT-PCR testing between January 1, 2021, and December 31, 2022. The index date was set as the day of the COVID-19 RT-PCR test.

Cohort 1 included children who had not received the COVID-19 vaccine before the index date and excluded those with a prior history of COVID-19 infection, asthma, neoplasms, or death before or up to one month after the PCR test. The objective of this cohort was to compare COVID-19-infected unvaccinated children with uninfected unvaccinated children. Cohort 2 consisted of children who had received at least one vaccine dose against COVID-19 before the index date, excluding those with prior COVID-19 infection, asthma, neoplasms, or death before the index date or up to one month after the PCR test. This analysis aimed to compare COVID-19 infected vaccinated children with uninfected vaccinated children.

Baseline characteristics and comorbidities were assessed from records dating one year prior to the index date. The baseline characteristics examined included age, gender, ethnicity, race, medical utilization (outpatient or inpatient services), socioeconomic status (problems related to housing and economic circumstances, factors influencing health status and contact with health services, problems related to education and literacy), lifestyle (nicotine dependence, alcohol related disorders), and comorbidities. Comorbidities comprised essential hypertension, diabetes mellitus, ischemic heart disease, mental, behavioral, and neurodevelopmental disorders, diseases of the nervous system, disorders of lipoprotein metabolism and other lipidemia, liver disease, chronic kidney disease, allergic rhinitis, gastro-esophageal reflux disease, atopic dermatitis, overweight and obesity, malnutrition, disorders of newborn related to short gestation and low birth weight.

Propensity Score Matching (PSM) is a statistical technique used to balance two groups in our study, addressing covariates that could bias the analysis results. We have selected several covariates for matching, including the baseline characteristics and comorbidities, which may be potential risk factors related to the outcome of interest. Balancing them between the study and comparison groups helps mitigate selection bias. On the TriNetX platform, selected demographics, ICD-10-PCS codes for procedures (medical utilization), and ICD-10 codes for comorbidities are set according to these criteria. The time window was set from 365 days to 1 day before the index event (PCR date). After identifying and matching these covariates using a 1:1 ratio, the balance of the matched samples is assessed using Standardized Mean Differences (SMDs). An SMD of < 0.1 indicated that there is no significant difference between the two groups.

To calculate the required sample size, we searched for literature related to our current study, we referenced a document by James P. Senter on asthma caused by SARS-CoV-2 virus [8]. We computed the required sample size analysis using a z-test in G*Power software [14]. The required sample sizes were 59,208 for both the COVID-19 and non-COVID-19 groups. Similarly, we used G*Power software with a z-test to analyze the statistical power.

### Outcomes

The primary outcome was the incidence of new-onset asthma or death, identified by ICD-10 codes in the database; the secondary outcome was the use of anti-asthmatic drugs or death. To mitigate the effects of competing risks and survivorship bias, we utilized a combination of ICD-10 codes and relevant medications to define composite outcomes. Given the potential confusion between post-COVID-19 respiratory symptoms and new-onset asthma, our study focuses on the risk of developing new-onset asthma within an 11-month period, from day 30 to day 365 following the index date.

### Subgroup analyses

Subgroup analyses were conducted on TriNetX separately to explore the differential risk of new-onset asthma based on sex (female, male), age groups (5–12 years, 13–18 years), and race (White, Black or African American, Asian, and other races), and disease severity. Severe COVID-19 is defined as requiring hospital inpatient services, ICU admission, or mechanical ventilation within one month of infection.

### Sensitivity analyses

The sensitivity analyses for the two cohorts utilized varying follow-up periods, from day 30 to day 730 post-index date, to test the reliability of the study’s outcomes. Additionally, our study used the COVID-19 Research Network within TriNetX,, which comprises 92 HCOs on the TriNetX platform. applying the same settings and propensity score matching (PSM) methodology as a sensitivity test to confirm the results’ global applicability.

### Statistical analyses

All procedures for data management and hazard ratio (HR) calculations, including 95% confidence intervals (CIs), were conducted within the TriNetX system. Baseline characteristics and comorbidities across all cohorts were evaluated, as detailed in Tables 1 and 2. Propensity score matching was employed for 1:1 matching based on age at index date, gender, ethnicity, race, medical utilization, socioeconomic status, lifestyle, and comorbidities to ensure balanced groups. Standardized mean differences (SMDs) of < 0.1 indicate that there is no significant difference between the two groups. Following this, HRs (95% CIs) were calculated using a Cox proportional hazards model, and p-values were determined via the log-rank test, with values below 0.05 considered statistically significant. Kaplan-Meier analysis estimated event rates over time, and the log-rank test examined differences in survival rates, offering a detailed statistical comparison of the groups.

**Table 1:** Baseline characteristics of Cohort 1, which never received the COVID-19 vaccine before the index date

	Before PSM					After PSM				
	COVID-19		Non COVID-19		SMD	COVID-19		Non COVID-19		SMD
**No. of individuals**	128,780		522,181			128,753		128,753		
**Age at index, years (mean ± SD)**	11.72 ± 4.09		11.05 ± 4.34		0.1590	11.72 ± 4.09		11.74 ± 4.10		0.0040
**Sex**										
Female	65,267	50.68%	263,322	50.43%	0.005074	65,248	50.68%	65,317	50.73%	0.001072
Male	61,026	47.39%	250,140	47.90%	0.010314	61,018	47.39%	60,915	47.31%	0.001602
**Ethnicity**										
Hispanic or Latino	19,225	14.93%	79,659	15.26%	0.009121	19,222	14.93%	19,103	14.84%	0.002597
Not Hispanic or Latino	85,153	66.12%	351,230	67.26%	0.024174	85,131	66.12%	85,022	66.04%	0.001788
Unknown Ethnicity	24,402	18.95%	91,292	17.48%	0.037983	24,400	18.95%	24,628	19.13%	0.00451
**Race**										
Asian	3810	2.96%	16,345	3.13%	0.009989	3810	2.96%	3860	3.00%	0.002284
White	72,317	56.16%	297,930	57.06%	0.018149	72,314	56.17%	72,296	56.15%	0.000282
Black or African American	27,983	21.73%	96,248	18.43%	0.08238	27,959	21.72%	28,032	21.77%	0.001374
Unknown Race	17,122	13.30%	72,945	13.97%	0.019636	17,122	13.30%	17,082	13.27%	0.000915
Other Race	6518	5.06%	34,922	6.69%	0.069205	6518	5.06%	6466	5.02%	0.001846
**Medical utilization**										
Office or Other Outpatient Services	51,659	40.11%	178,054	34.10%	0.124774	51,633	40.10%	51,259	39.81%	0.00593
Hospital Inpatient and Observation Care Services	2389	1.86%	5619	1.08%	0.064862	2365	1.84%	2053	1.60%	0.018662
**Social economic status**										
Problems related to housing and economic circumstances	212	0.17%	933	0.18%	0.003395	212	0.17%	161	0.13%	0.010415
Factors influencing health status and contact with health services	67,476	52.40%	232,358	44.50%	0.158547	67,449	52.39%	67,261	52.24%	0.002923
Problems related to education and Literacy	358	0.28%	1210	0.23%	0.009178	356	0.28%	278	0.22%	0.012224
**Lifestyle**										
Nicotine dependence	345	0.27%	1022	0.20%	0.01501	345	0.27%	301	0.23%	0.006832
Alcohol related disorders	89	0.07%	484	0.09%	0.008293	89	0.07%	68	0.05%	0.006608
**Comorbidities**										
Essential (primary) hypertension	890	0.69%	2103	0.40%	0.039107	879	0.68%	754	0.59%	0.01223
Diabetes mellitus	826	0.64%	3574	0.68%	0.005303	826	0.64%	683	0.53%	0.014552
Ischemic heart diseases	48	0.04%	144	0.03%	0.005386	48	0.04%	37	0.03%	0.004703
Endocrine, nutritional and metabolic diseases	10,788	8.38%	35,971	6.89%	0.05608	10,769	8.36%	10,082	7.83%	0.019561
Mental, Behavioral and Neurodevelopmental disorders	17,811	13.83%	64,692	12.39%	0.042728	17,799	13.82%	17,303	13.44%	0.011227
Diseases of the nervous system	10,232	7.95%	40,305	7.72%	0.008439	10,226	7.94%	9537	7.41%	0.020104
Disorders of lipoprotein metabolism and other lipidemias	909	0.71%	2968	0.57%	0.017278	907	0.70%	802	0.62%	0.010044
Diseases of liver	442	0.34%	1425	0.27%	0.012691	442	0.34%	288	0.22%	0.022498
Chronic kidney disease (CKD)	291	0.23%	766	0.15%	0.018383	288	0.22%	221	0.17%	0.011716
Allergic rhinitis, unspecified	3694	2.87%	8634	1.65%	0.081802	3673	2.85%	3576	2.78%	0.004555
Gastro-esophageal reflux disease without esophagitis	1954	1.52%	6498	1.24%	0.023389	1950	1.52%	1650	1.28%	0.019847
Atopic dermatitis	1421	1.10%	4107	0.79%	0.032762	1417	1.10%	1260	0.98%	0.012022
Overweight and obesity	4855	3.77%	14,787	2.83%	0.052532	4848	3.77%	4755	3.69%	0.003812
Malnutrition	537	0.42%	1695	0.33%	0.015201	535	0.42%	381	0.30%	0.020091
Disorders of newborn related to short gestation and low birth weight	228	0.18%	844	0.16%	0.003749	228	0.18%	172	0.13%	0.011044

SD, standard deviation; SMD, standardized mean difference; PSM: matching factors included age at index, sex, ethnicity, race, medical utilization, social economic status, and comorbidities

**Table 2:** Baseline characteristics of Cohort 2, which received the COVID-19 vaccine before the index date

Cohort 2	Before PSM					After PSM				
	COVID-19		Non COVID-19		SMD	COVID-19		Non COVID-19		SMD
**No. of individuals**	23,590		77,708			23,497		23,497		
**Age at index, years (mean ± SD)**	12.21 ± 3.94		11.82 ± 4.21		0.09479	12.20 ± 3.94		12.22 ± 3.98		0.0046451
**Sex**										
Female	12,237	51.87%	39,906	51.35%	0.0104033	12,180	51.84%	12,181	51.84%	8.517E-05
Male	10,726	45.47%	35,468	45.64%	0.0034987	10,690	45.50%	10,697	45.53%	0.0005982
**Ethnicity**										
Hispanic or Latino	4862	20.61%	14,008	18.03%	0.0654858	4844	20.62%	4703	20.02%	0.0149149
Not Hispanic or Latino	14,580	61.81%	48,923	62.96%	0.0237748	14,512	61.76%	14,546	61.91%	0.0029786
Unknown Ethnicity	4148	17.58%	14,777	19.02%	0.0370497	4141	17.62%	4248	18.08%	0.0118917
**Race**										
Asian	1895	8.03%	6281	8.08%	0.0018281	1891	8.05%	1958	8.33%	0.0103985
White	11,911	50.49%	39,944	51.40%	0.0182231	11,883	50.57%	11,881	50.56%	0.0001702
Black or African American	4525	19.18%	12,582	16.19%	0.0784361	4475	19.05%	4510	19.19%	0.0037879
Unknown Race	2770	11.74%	10,060	12.95%	0.0365972	2770	11.79%	2705	11.51%	0.0086225
Other Race	2119	8.98%	7697	9.91%	0.031546	2112	8.99%	2092	8.90%	0.0029823
**Medical utilization**										
Office or Other Outpatient Services	13,640	57.82%	36,273	46.68%	0.224477	13,547	57.65%	13,526	57.57%	0.0018085
Hospital Inpatient and Observation Care Services	647	2.74%	955	1.23%	0.1086608	570	2.43%	544	2.32%	0.0072736
**Social economic status**										
Problems related to housing and economic circumstances	79	0.34%	369	0.48%	0.0220432	79	0.34%	48	0.20%	0.0254151
Factors influencing health status and contact with health services	18,385	77.94%	51,846	66.72%	0.25271	18,292	77.85%	18,316	77.95%	0.0024617
Problems related to education and Literacy	132	0.56%	315	0.41%	0.0222545	130	0.55%	109	0.46%	0.0125645
**Lifestyle**										
Nicotine dependence	47	0.20%	122	0.16%	0.0100173	47	0.20%	41	0.17%	0.0059065
Alcohol related disorders	15	0.06%	67	0.09%	0.0082733	15	0.06%	10	0.04%	0.0092285
**Comorbidities**										
Essential (primary) hypertension	209	0.89%	654	0.84%	0.0047933	237	1.01%	209	0.89%	0.0122907
Diabetes mellitus	209	0.89%	654	0.84%	0.0047933	206	0.88%	151	0.64%	0.0269608
Ischemic heart diseases	10	0.04%	18	0.02%	0.010622	10	0.04%	10	0.04%	0
Endocrine, nutritional and metabolic diseases	3383	14.34%	8304	10.69%	0.1106244	3316	14.11%	2993	12.74%	0.0403296
Mental, Behavioral and Neurodevelopmental disorders	5130	21.75%	14,395	18.52%	0.0804122	5071	21.58%	4902	20.86%	0.0175912
Diseases of the nervous system	2811	11.92%	6630	8.53%	0.1118753	2737	11.65%	2549	10.85%	0.025325
Disorders of lipoprotein metabolism and other lipidemias	402	1.70%	1031	1.33%	0.0308918	396	1.69%	323	1.38%	0.0253134
Diseases of liver	158	0.67%	293	0.38%	0.0405754	144	0.61%	137	0.58%	0.0038642
Chronic kidney disease (CKD)	154	0.65%	238	0.31%	0.0501792	132	0.56%	110	0.47%	0.0130814
Allergic rhinitis, unspecified	1257	5.33%	2144	2.76%	0.1307196	1217	5.18%	1201	5.11%	0.0030823
Gastro-esophageal reflux disease without esophagitis	584	2.48%	1019	1.31%	0.0855038	532	2.26%	442	1.88%	0.026888
Atopic dermatitis	558	2.37%	1291	1.66%	0.0501419	542	2.31%	482	2.05%	0.0174908
Overweight and obesity	1714	7.27%	4047	5.21%	0.0851734	1699	7.23%	1605	6.83%	0.015648
Malnutrition	179	0.76%	366	0.47%	0.0368219	165	0.70%	138	0.59%	0.0143571
Disorders of newborn related to short gestation and low birth weight	53	0.23%	123	0.16%	0.0151862	51	0.22%	40	0.17%	0.010649

SD, standard deviation; SMD, standardized mean difference; PSM: matching factors included age at index, sex, ethnicity, race, medical utilization, social economic status, and comorbidities

To address potential biases and confounders, Propensity Score Matching (PSM) was utilized to control for confounding by pairing participants with similar likelihoods of exposure. This method aimed to mitigate bias, and the effectiveness of the matching process was evaluated by examining any residual imbalances. Several sensitivity analyses were conducted to ensure robustness. These included using different networks, such as the COVID-19 Research Network, which consists of 92 healthcare organizations on the TriNetX platform. Additionally, the follow-up period was extended to 730 days, and analyses were stratified by severe COVID-19 infection to explore the impact of geography and infection severity. Model diagnostics were performed to assess the robustness of the statistical outcomes. To address survival and competition biases, each outcome of interest was combined with death in the analysis. This method provided a more accurate representation of the overall effects and minimized potential distortions in the results.

### Ethical considerations

This retrospective study has received approval from the Institutional Review Board of Chung Shan Medical University Hospital (IRB reference number CS1-23211). In conducting research using the TriNetX database, we maintained stringent adherence to ethical guidelines. All data were de-identified to protect patient privacy, and the study involved no direct contact with patients. Furthermore, given that the research relied on pre-existing anonymized data, a waiver for patient informed consent was obtained. We are committed to upholding high ethical standards throughout all stages of the research, ensuring both the integrity of the study and the safety of participants.

## Results

### Baseline characteristics of the study individuals

Figure 1 displays the flowchart for cohort selection and construction, with baseline characteristics of the subjects summarized in Tables 1 and 2. Before Propensity Score Matching (PSM), significant differences were observed between the COVID-19 and non-COVID-19 groups in terms of age at the index date, medical utilization, factors influencing health status, and contact with health services in Cohort 1, as well as medical utilization, factors influencing health status, endocrine, nutritional and metabolic diseases, and allergic rhinitis in Cohort 2. After matching, the study identified an equal number of children in both the COVID-19 and non-COVID-19 groups: 128,753 in Cohort 1 and 23,497 in Cohort 2. A standardized mean difference (SMD) of less than 0.1 indicated minimal and negligible differences in covariates between the two groups. In the COVID-19 infected children group, the mean age at the index date was approximately 11.72 ± 4.09 years in Cohort 1 and 12.20 ± 3.94 years in Cohort 2 at the index date. The majority were female (Cohort 1, 50.68%; Cohort 2, 51.84%) and predominantly of White race (Cohort 1, 56.17%; Cohort 2, 50.57%).

### Incidence of new-onset asthma in the COVID-19 and non-COVID-19 groups

Regardless of vaccination status, the COVID-19 infected group exhibited a higher incidence of outcomes compared to the non-infected group. The incidence of new-onset asthma or death was illustrated in the COVID-19 infected group compared to non-infected group across various cohorts (see Table 3). In Cohort 1, 4.7% of COVID-19 infected unvaccinated children developed new-onset asthma or death, compared to 2.0% in their non-COVID-19 counterparts within a year. For Cohort 2, COVID-19 infected vaccinated children exhibited an 8.3% incidence of new-onset asthma or death, which was higher than the 3.1% observed in those who were not infected. The COVID-19 infected group exhibited a 126% increased risk (HR = 2.26; 95% CI = 2.158–2.367) of developing new-onset asthma or death compared to the non-infected group in Cohort 1 and 174% increased risk (HR = 2.745; 95% CI = 2.521–2.99) in Cohort 2. For the secondary outcome, in Cohort 1, 21.1% of COVID-19-infected unvaccinated children were either prescribed anti-asthmatic drugs or died, compared to 16.7% in the non-COVID-19 group (HR = 1.236; 95% CI = 1.214–1.259). In contrast, Cohort 2 exhibited a slightly higher incidence, with 28.3% of COVID-19-infected vaccinated children experiencing the same outcomes compared to 21.7% in their non-infected counterparts (HR = 1.375; 95% CI = 1.325–1.426). Regarding the composite outcomes, Cohort 1 observed a 22.0% incidence among COVID-19-infected unvaccinated children versus 17.3% in the non-infected group (HR = 1.218; 95% CI = 1.196–1.24). Cohort 2 exhibited a further elevated incidence of 27.5% in COVID-19-infected vaccinated children compared to 21.7% in the non-infected group (HR = 1.342; 95% CI = 1.293–1.392). These results show that children who are infected with COVID-19 after being vaccinated against COVID-19 are at a higher risk of developing asthma than children who are infected with COVID-19 who are not vaccinated against COVID-19.

**Table 3:** HR and 95% CIs for the risk of outcomes in Cohort 1 (*n* = 128,753) and Cohort 2 (*n* = 23,497)

	Cohort 1 Never received COVID-19 vaccine before the index date		Cohort 2 Received COVID-19 vaccine before the index date	
	Patients with outcome (%)	HR (95% CI)	Patients with outcome (%)	HR (95% CI)
**Asthma or death**				
Non-COVID-19	2568 (2.0%)	Reference	726 (3.1%)	Reference
COVID-19	6026 (4.7%)	2.26 (2.158,2.367)	1941 (8.3%)	2.745 (2.521,2.99)
**Any anti-asthmatic drugs or death**				
Non-COVID-19	21,544 (16.7%)	Reference	5089 (21.7%)	Reference
COVID-19	27,232 (21.1%)	1.236 (1.214,1.259)	6670 (28.3%)	1.375 (1.325,1.426)
**Asthma or anti-asthmatic drugs or death**				
Non-COVID-19	22,290 (17.3%)	Reference	5090 (21.7%)	Reference
COVID-19	28,275 (22.0%)	1.218 (1.196,1.24)	6451 (27.5%)	1.342 (1.293,1.392)

*Hazard ratio (HR) and 95% CI are provided, demonstrating outcomes among COVID-19 infected individuals versus non-infected counterparts

*The achieved power of this study is calculated to be approximately 1 for all tests.

Initially, to address survival bias and competition bias, we combined each outcome of interest with death for the analysis. We independently analyzed ‘death’ and recreated the table, which is presented in Table S2. We found that COVID-19 infection had a non-significant statistical impact on child mortality within one year. However, the other outcomes of interest, including new-onset asthma, the use of asthma medication, and the composite outcome, all showed an increased risk.

**Fig. 2:**
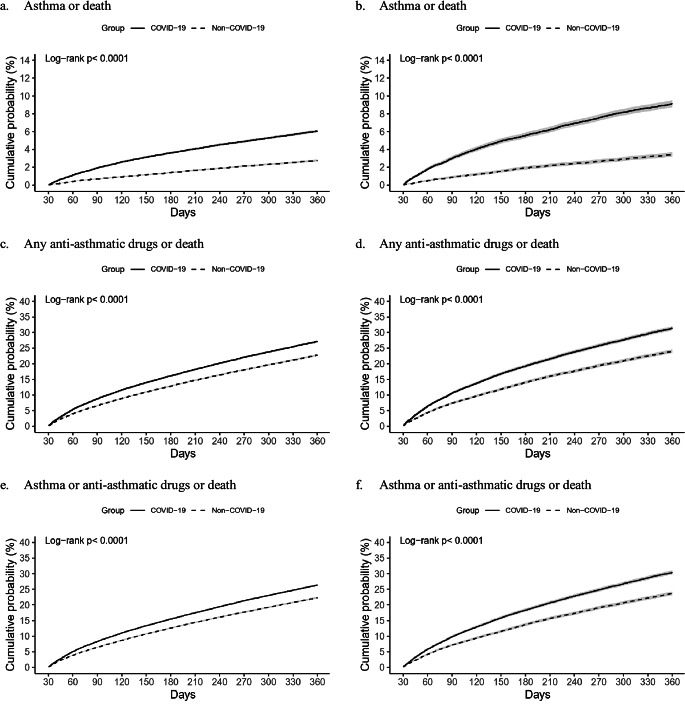
Kalpan-Meier curves of propability(%) of outcomes comparing COVID-19 group,from Day 30 to Day 365 post the Index Date.Panels(a),(c)and (e) depiet, Cohert 1: Panels (b),(d) and (f) depiet Cohort 2

Figure 2 presents Kaplan-Meier curves for the incidence of new-onset asthma, the use of any anti-asthmatic drugs, and a composite outcome, all curves show a trend of increasing incidence over time. The log-rank test revealed a significant difference between the COVID-19 group and non-COVID-19 group (*p* < 0.001). Supplementary Table S1 shows the survival probability calculated on TriNetX on the 30th, 60th, 90th, 180th, and 365th days after the index date in Cohort 1 and Cohort 2.

### Subgroup analysis

We further examined the risk of incident asthma in subgroups based on sex, age, and race, as detailed in Tables 4 and 5. Subgroup analysis revealed a higher risk of new-onset asthma or death among males, children aged 5–12 years, and those who are Black or African American, regardless of vaccination status. Specifically, for Cohort 1, the HR for asthma or death among males was 2.308 (95% CI = 2.168–2.456), compared to females with an HR of 2.212 (95% CI = 2.071–2.364). For children aged 5–12 years, the HR was 1.883 (95% CI = 1.786–1.985), and for Black or African American individuals, the HR was 2.204 (95% CI = 2.029–2.393). In Cohort 2, similar trends were observed. The HR for asthma or death among males was 2.747 (95% CI = 2.463–3.068), and for children aged 5–12 years, the HR was 2.517 (95% CI = 2.272–2.788). Black or African American individuals in this cohort had an HR of 2.948 (95% CI = 2.512–3.46) for asthma or death.

**Table 4:** Subgroup analysis of Cohort 1, which never received the COVID-19 vaccine before the index date

	Asthma or death			Anti-asthmatic drug or death			Asthma or anti-asthmatic drug or death		
	Patients with outcome		HR (95% CI)	Patients with outcome		HR (95% CI)	Patients with outcome		HR (95% CI)
	COVID	Non-COVID		COVID	Non-COVID		COVID	Non-COVID	
**Sex**									
Female (*n* = 72,936)	2911	1260	2.212 (2.071,2.364)	14,993	11,707	1.248 (1.218,1.278)	14,579	11,479	1.233 (1.203,1.264)
Male (*n* = 68,180)	3357	1409	2.308 (2.168,2.456)	13,768	10,848	1.246 (1.215,1.278)	13,302	10,593	1.228 (1.197,1.26)
**Age**									
5–12 (*n* = 77,744)	4011	2104	1.883 (1.786,1.985)	16,486	13,744	1.198 (1.171,1.226)	15,985	13,454	1.182 (1.156,1.21)
13–18 (*n* = 66,211)	2435	1379	1.731 (1.62,1.849)	12,892	11,487	1.105 (1.078,1.133)	12,482	11,181	1.097 (1.069,1.125)
**Race**									
Black or African American (*n* = 28,695)	1858	815	2.204 (2.029,2.393)	6304	5169	1.184 (1.141,1.228)	6038	5017	1.162 (1.119,1.206)
White (*n* = 83,432)	3222	1420	2.178 (2.046,2.318)	17,142	13,606	1.228 (1.201,1.256)	16,675	13,369	1.212 (1.185,1.24)
Asian (*n* = 3912)	111	58	1.849 (1.346,2.54)	723	594	1.205 (1.081,1.343)	708	584	1.199 (1.075,1.338)
Other race (*n* = 8177)	363	158	2.138 (1.773,2.577)	1534	1190	1.214 (1.125,1.309)	1481	1175	1.182 (1.095,1.276)

*Hazard ratio (HR) and 95% CI are provided, demonstrating outcomes among COVID-19 infected individuals versus non-infected counterparts

**Table 5:** Subgroup analysis of Cohort 2, which received the COVID-19 vaccine before the index date

	Asthma or death			Anti-asthmatic drug or death			Asthma or anti-asthmatic drug or death		
	Patients with outcome		HR (95% CI)	Patients with outcome		HR (95% CI)	Patients with outcome		HR (95% CI)
	COVID	Non-COVID		COVID	Non-COVID		COVID	Non-COVID	
**Sex**									
Female (*n* = 15,584)	1215	515	2.405 (2.172,2.666)	4239	3265	1.347 (1.287,1.409)	4049	3211	1.315 (1.256,1.377)
Male (*n* = 13,605)	1162	432	2.747 (2.46,3.068)	3706	2860	1.346 (1.282,1.413)	3537	2811	1.295 (1.233,1.361)
**Age**									
5–12 (*n* = 14,406)	1262	514	2.517 (2.272,2.788)	3952	3064	1.346 (1.284,1.411)	3807	3010	1.312 (1.241,1.376)
13–18 (*n* = 15,551)	1175	547	2.188 (1.977,2.422)	4186	3262	1.327 (1.267,1.389)	4007	3194	1.288 (1.23,1.35)
**Race**									
Black or African American (*n* = 4829)	576	203	2.948 (2.512,3.46)	1539	1265	1.267 (1.176,1.364)	1475	1244	1.224 (1.135,1.319)
White (*n* = 16,840)	1240	507	2.471 (2.228,2.74)	4510	3476	1.331 (1.273,1.391)	4345	3409	1.298 (1.241,1.358)
Asian (*n* = 2051)	108	50	2.261 (1.617,3.162)	389	356	1.15 (0.996,1.328)	373	349	1.12 (0.968,1.296)
Other race (*n* = 2517)	219	83	2.644 (2.053,3.404)	698	535	1.322 (1.181,1.48)	662	532	1.248 (1.114,1.399)

*Hazard ratio (HR) and 95% CI are provided, demonstrating outcomes among COVID-19 infected individuals versus non-infected counterparts

When restricting the analysis to severe COVID-19 infections, defined by hospital inpatient stays, intensive care unit stays, or mechanical ventilation within one month after infection, there was a significantly higher risk of asthma during the follow-up period for both Cohort 1 (HR = 3.743; 95% CI = 2.975–4.71) and Cohort 2 (HR = 2.964; 95% CI = 2.046–4.293) (Table S3).

### Sensitivity analysis

In the sensitivity analysis of two cohorts, when extending the follow-up time to 2 years, the COVID-19 group exhibited a consistent trend of higher risk for new-onset asthma compared with the non-COVID-19 group. Moreover, this study utilized the COVID-19 Research Network on TriNetX and yielded results that were consistent with the primary approach (Table S4, Figure S1).

## Discussion

This large retrospective cohort study utilized TriNetX, a real-world database, to evaluate the impact of COVID-19 infection and vaccination on the incidence of new-onset asthma in children. Regardless of vaccination status, groups infected with COVID-19 exhibited a significantly increased incidence of new-onset asthma, anti-asthmatic drug use and composite outcomes compared to those not infected, from the 30th to the 365th day following the index date, over a period of 11 months. The subgroup analysis was consistent across gender, age, and racial groups.

The link between viral infections and asthma exacerbation is well-established, but the association with new-onset asthma, particularly in the context of COVID-19, is a relatively novel area of research. Our findings support the increasing evidence that COVID-19 may trigger asthma development. This corresponds to findings from research on other respiratory viruses, such as rhinovirus (RV) and respiratory syncytial virus (RSV), which are recognized for triggering asthma in individuals with a genetic predisposition. In a Finnish study, rhinoviruses were also identified as significant inducers of wheezing [15]. A systematic review of 28 articles revealed an increased risk of asthma following hospitalization for RSV in infancy and childhood [16]. Also, the findings of a meta-analysis suggested that experiencing wheezing illness caused by RV during the first 3 years of life was linked to the later development of wheezing or asthma [17]. Rhinoviruses infection promotes the secretion of cytokines from epithelial cells, such as IL-6, IL-8, IL-11, and GM-CSF, which may trigger the onset of asthma [18–21]. In a large, population-based, birth cohort study (INSPIRE), not being infected with RSV in the first year of life was associated with a 26% lower risk of asthma at age 5 compared to those who were infected with RSV (adjusted RR = 0.74, 95% CI = 0.58–0.94, *p* = 0.014).^22^ Furthermore, RSV infection in infants has been shown to affect T-cell memory responses and airway epithelium development [23]. The interaction of viral virulence factors, individual genetic susceptibilities, and environmental factors—such as exposure to the airway microbiome—plays a significant role in the aggravation of wheezing illnesses and the increased risk of asthma development [24, 25]. 

Some literature has indicated the impact of COVID-19 on individuals with asthma following infection. The interaction of the virus with the ACE2 receptor, which is variably expressed in asthma patients, may influence the severity of COVID-19 and could potentially exacerbate asthma symptoms [26]. One study highlighted the importance of host factors in both asthma and COVID-19, discovering shared genetic and molecular pathways in the two conditions through the analysis of bronchoalveolar lavage fluid, suggests that COVID-19 may contribute to the exacerbation or onset of asthma in affected patients [27]. 

To date, there are few studies that explore the issue of new-onset asthma caused by COVID-19 infection. A nationwide, population-based cohort study emphasized that adults who were infected with COVID-19 had a higher incidence of new-onset asthma compared to those uninfected [5]. This suggests a potential risk factor for developing asthma following COVID-19 infection. However, earlier research has been limited by smaller sample sizes and relatively brief observation periods.

A review article found that many viruses, particularly single-stranded RNA viruses, can cause asthma [28]. SARS-CoV, also a single-stranded RNA virus, may have similar effects or mechanism. Research shows that certain viruses can cause epithelial cells to produce pro-Th2 cytokines such as IL-25 and IL-33, activating ILC2s, DCs, and Th2 cells, thereby promoting allergic inflammation [29, 30]. It has been reported that serum IL-33 levels are elevated in COVID-19 patients. This cytokine increases neutrophil activity via the IL-33/ST2 pathway and inhibits IFN-I secretion by pDCs. Additionally, IL-33 disrupts the Th17/Treg balance in the lungs, leading to cytokine storms and immune damage. IL-33 also causes hyperactivation of ILC2s, differentiation of M2 macrophages, and secretion of TGF-β and IL-13 [31]. Common coronavirus infections in children trigger host responses similar to those induced by other viruses, such as increased expression of IL6 and ACE2 [32]. COVID-19 infection can also trigger an imbalanced immune response, characterized by a cytokine storm and overproduction of pro-inflammatory cytokines [33–35]. Additionally, the infection may impair mucociliary clearance, a vital respiratory defense, leading to mucus and pathogen accumulation in the airways [33]. In COVID-19 patients, there’s a marked increase in IL-4 expression and a rise in M2 macrophages, suggesting a significant Th2 immune response and anti-inflammatory activity that potentially leads to airway remodeling [36]. Unlike the limited Th1/Th17 response, the strong Th2 response in SARS-CoV-2 induced lung injury suggests a mechanism that favors airway remodeling [36]. 

In the United States, the Pfizer-BioNTech COVID-19 vaccine was authorized for those 16 and older on December 11, 2020, and approved by the FDA on August 23, 2021. Its use expanded to 12–15 year-olds on May 10, 2021, and to children 5–11 on October 29, 2021. By July 6, 2022, CDC data showed 37% of 5–11 year-olds and 70% of 12–17 year-olds had received at least one vaccine dose. Two case reports have indicated that the SARS-CoV-2 vaccine could potentially exacerbate asthma, suggesting that repeated vaccinations might be a risk factor for severe asthma flare-ups. This association is thought to involve inflammatory and immunological responses [37, 38]. 

To date, there have been several cohort studies that have linked COVID-19 infection in both adults and children. Kim utilized the Korean National Health Insurance claim-based database to design three cohorts for adults and found that 1.6% of the COVID-19 group and 0.7% of the non-infected group developed asthma during the 6-month observation period. The COVID-19 cohort faced a higher risk of new-onset asthma (aHR 2.14; 95% CI 1.88–2.45) than matched controls, aligning with our findings. Kim noted that vaccinated infected individuals had a lower asthma risk compared to their unvaccinated counterparts (aHR 0.82; 95% CI 0.76–0.89). However, vaccination did not reduce asthma risk in those uninfected by COVID-19 (aHR 0.95; 95% CI 0.87–1.04) [6]. A study with cohorts from South Korea, Japan, and the UK shows a significant rise in allergic diseases like asthma and allergic rhinitis after COVID-19, persisting over six months and worsening with disease severity. Vaccination with at least two COVID-19 doses provided protection [7]. As for children, there is limited and inconclusive literature on the risk of asthma following COVID-19 infection in children. A small sample size retrospective chart review revealed that most children diagnosed with COVID-19 had normal spirometry and plethysmography results [39]. Furthermore, a retrospective cohort study using EHR data from a single institution within the Children’s Hospital of Philadelphia Care Network found no association between SARS-CoV-2 PCR positivity and new asthma diagnoses in children aged 1–16 over an 18-month follow-up period (HR: 0.96; *P* = 0.79).^8^ Interestingly, another study indicated that an asthma diagnosis was negatively associated with COVID-19-related hospitalization in children [9]. To date, no strong evidence has been published in the literature to suggest a positive or negative association between COVID-19 and new-onset asthma in children.

However, our study reveals contrasting results, indicating that children vaccinated against COVID-19 who contracted the virus had an increased risk of developing new-onset asthma (HR = 2.745, 95% CI = 2.521, 2.99). This could be attributed to various factors, including immune response to the vaccine, the interaction between the vaccine and the virus, or other unrelated environmental or genetic factors.

Our subgroup analysis identified higher risks among younger age groups and Black or African American individuals. Following COVID-19 infection, infants developed a strong mucosal immune response marked by inflammatory cytokines, IFN-α, and Th-17 and neutrophil markers like IL-17, IL-8, and CXCL1 [40]. This response led to further respiratory inflammation and associated symptoms, which may explain why younger age groups have a higher risk of developing asthma. Regarding ethnicity, a large database analysis of adults across three national cohorts (UK, South Korea, Japan) found consistent results, indicating that post-COVID-19 effects on allergic diseases are not influenced by race [7]. However, the above study focused on adults and mainly included data from Asian countries. For children, we speculate that the immune response of Black individuals after COVID-19 infection may be influenced by genetics, socioeconomic status, and health disparities, but more research is needed to confirm this.

The strengths of our study include its use of a large, nationwide, population-based cohort and rigorous statistical analyses, including propensity score matching, to reduce selection bias. Moreover, our research provides valuable insights into the association between COVID-19 vaccination/infection and the incidence of new-onset asthma in children, an area currently seldom explored.

Our retrospective cohort analysis faced several limitations. The generalizability of our findings is affected by regional bias and variations in healthcare systems within the TriNetX database, which primarily includes U.S. data. This limits the applicability of our results to regions with different healthcare systems and demographics. Additionally, missing data is an issue, as some asthmatic patients, particularly children, may not have sought care from HCOs in the TriNetX network. Confounding bias is another concern; although we used Propensity Score Matching (PSM) to control for biases, unmeasured confounders such as family history of asthma and environmental exposures may still influence the results. Misclassification bias was mitigated by restricting COVID-19 diagnoses to PCR-positive cases. However, relying solely on ICD-10 codes without pulmonary function tests can reduce diagnostic accuracy. Using asthma medications as a secondary outcome helped enhance diagnostic precision. The study also overlooked the impact of COVID-19 variants and vaccination timing or type, which could significantly affect immune responses and asthma risk. Despite these efforts, the observational nature of our study limits the ability to confirm causality.

## Conclusion

This study identifies a significant link between COVID-19 infection and an increased incidence of new-onset asthma in children, with higher risk in vaccinated individuals. These findings underscore the need for ongoing monitoring and targeted healthcare strategies to mitigate long-term respiratory impacts in children.

Public health actions should focus on enhanced surveillance of respiratory health post-COVID-19 infection, regardless of vaccination status. Tools like the ISAAC (International Study of Asthma and Allergies in Childhood) questionnaire, the GINA (Global Initiative for Asthma) symptom control tool, and the PACS (Pediatric Asthma Control Score) can help identify high-risk children early.

Clinicians should be aware of new-onset asthma in pediatric patients post-COVID-19 and implement preventive measures and early interventions. This includes providing asthma education for parents, educators, and children, and offering professional asthma management plans with personalized medication guidance and environmental control advice. Healthcare systems should establish asthma databases to track incidence, treatment outcomes, and health results to guide policymaking. As the virus continues to mutate, it is essential to closely monitor whether the long-term prevalence of COVID-19 affects asthma risk in children.

Further research is necessary to explore causal pathways linking COVID-19 to asthma development, refine public health guidelines, and better protect children’s health in future pandemics.

## Electronic supplementary material

Below is the link to the electronic supplementary material.

Supplementary Material 1

Supplementary Material 2

## Data Availability

The de-identified data in the TriNetX federated network database can only be accessed by researchers who are either part of the network or have a collaboration agreement with TriNetX. We utilized data from the TriNetX database under a no-cost collaboration agreement between Chung Shan Medical University Hospital and TriNetX. Under this agreement, we accessed de-identified data in accordance with the agreements and institutional approvals already in place between TriNetX and their partner institutions. The data utilized in this study can be obtained from TriNetX by future researchers who establish a collaboration with TriNetX.
