# Supplementary material for: The association between COVID-19 vaccine/infection and new-onset asthma in children - based on the global TriNetX database
Source: Infection. 2024 Jun 21;53(1):125–37. doi: 10.1007/s15010-024-02329-3 (PMC11825542; doi:10.1007/s15010-024-02329-3)
Supplement: Supplementary file 1 — Supplementary Material 1 [file 15010_2024_2329_MOESM1_ESM.docx]

**Supplementary**

**Table S1: Survival probability and Hazard ratio (95% CI) for the risk of outcomes in (a) Cohort 1 (n=128,753) and (b) Cohort 2 (n=23,497)**

1. Cohort 1, which never received the COVID-19 vaccine before the index date.

|  | **Survival probability (%) since index date** | | | | |  |
| --- | --- | --- | --- | --- | --- | --- |
|  | 30 days | 60 days | 90 days | 180 days | 365 days | HR (95% CI) |
| **Asthma or death** |  |  |  |  |  |  |
| Non-COVID-19 | 99.99 | 99.59 | 99.32 | 98.60 | 97.19 | Reference |
| COVID-19 | 99.96 | 98.87 | 98.06 | 96.39 | 93.88 | 2.26 (2.158,2.367) |
| **Any anti-asthmatic drugs or death** |  |  |  |  |  |  |
| Non-COVID-19 | 99.82 | 95.98 | 93.37 | 87.14 | 76.90 | Reference |
| COVID-19 | 99.75 | 94.58 | 91.19 | 83.83 | 72.53 | 1.236 (1.214,1.259) |
| **Asthma or anti-asthmatic drugs or death** |  |  |  |  |  |  |
| Non-COVID-19 | 99.82 | 96.09 | 93.56 | 87.43 | 77.35 | Reference |
| COVID-19 | 99.77 | 94.92 | 91.68 | 84.52 | 73.34 | 1.218 (1.196,1.24) |

1. Cohort 2, which received the COVID-19 vaccine before the index date.

|  | **Survival probability (%) since index date** | | | | |  |
| --- | --- | --- | --- | --- | --- | --- |
|  | 30 days | 60 days | 90 days | 180 days | 365 days | HR (95% CI) |
| **Asthma or death** |  |  |  |  |  |  |
| Non-COVID-19 | 99.98 | 99.51 | 99.12 | 98.10 | 96.50 | Reference |
| COVID-19 | 099.94 | 98.20 | 96.98 | 94.43 | 90.80 | 2.745 (2.521,2.99) |
| **Any anti-asthmatic drugs or death** |  |  |  |  |  |  |
| Non-COVID-19 | 99.75 | 95.64 | 92.59 | 85.97 | 75.66 | Reference |
| COVID-19 | 99.73 | 93.52 | 89.41 | 80.74 | 68.34 | 1.375 (1.325,1.426) |
| **Asthma or anti-asthmatic drugs or death** |  |  |  |  |  |  |
| Non-COVID-19 | 99.77 | 95.81 | 92.84 | 86.31 | 76.00 | Reference |
| COVID-19 | 99.76 | 94.16 | 90.18 | 81.66 | 69.34 | 1.342 (1.293,1.392) |

Hazard ratio (HR) and 95% CI are provided, demonstrating outcomes among COVID-19 infected individuals versus non-infected counterparts

**Table S2: HR and 95% CIs for the risk of outcomes in Cohort 1 (n=159,357) and Cohort 2 (n=32,088)**

|  | **Cohort 1**  **Never received COVID-19 vaccine before the index date** | |  | **Cohort 2**  **Received COVID-19 vaccine before the index date** | |
| --- | --- | --- | --- | --- | --- |
|  | Patients with outcome (%) | HR (95% CI) |  | Patients with outcome (%) | HR (95% CI) |
| **Asthma** |  |  |  |  |  |
| Non-COVID-19 | 2988 (1.9%) | Reference |  | 767 (2.4%) | Reference |
| COVID-19 | 7207 (4.5%) | 2.372 (2.273,2.476) |  | 2386 (7.4%) | 3.198 (2.948,3.469) |
| **Any anti-asthmatic drugs** |  |  |  |  |  |
| Non-COVID-19 | 23246 (14.6%) | Reference |  | 5956 (18.6%) | Reference |
| COVID-19 | 30181 (18.9%) | 1.296 (1.274,1.318) |  | 8156 (25.4%) | 1.435 (1.388,1.484) |
| **Death** |  |  |  |  |  |
| Non-COVID-19 | 140 (0.1%) | Reference |  | 188 (0.6%) | Reference |
| COVID-19 | 169 (0.1%) | 1.164 (0.930,1.456) |  | 166 (0.5%) | 0.880 (0.714,1.084) |
| **Asthma or anti-asthmatic drugs or death** |  |  |  |  |  |
| Non-COVID-19 | 22423 (14.1%) | Reference |  | 5777 (18.0%) | Reference |
| COVID-19 | 28538 (17.9%) | 1.263 (1.241,1.286) |  | 7678 (23.9%) | 1.382 (1.335,1.430) |

*Hazard ratio (HR) and 95% CI are provided, demonstrating outcomes among COVID-19 infected individuals versus non-infected counterparts

*The achieved power of this study is calculated to be approximately 1 for all tests.

**Table S3: Risk of new-onset asthma or death in individuals exposed to severe COVID-19 infection compared to those not infected with COVID-19 in Cohort 1 (n=3081) and Cohort 2 (n=612). Severe COVID-19 infection is characterized by hospitalization, ICU admission, or mechanical ventilation within one month post-infection.**

|  | **Cohort 1**  **Never received COVID-19 vaccine before the index date** | |  | **Cohort 2**  **Received COVID-19 vaccine before the index date** | |
| --- | --- | --- | --- | --- | --- |
|  | Patients with outcome | HR (95% CI) |  | Patients with outcome | HR (95% CI) |
| **Asthma or death** |  |  |  |  |  |
| Non-COVID | 94 | Reference |  | 38 | Reference |
| Severe COVID-19 | 322 | 3.743 (2.975,4.71) |  | 106 | 2.964 (2.046,4.293) |

Hazard ratio (HR) and 95% CI are provided, demonstrating outcomes among severe COVID-19 infected individuals versus non-infected counterparts

**Table S4: Sensitivity analysis of the risk of new-onset asthma or death of Cohort 1 and Cohort 2**

1. Follow from Day 30 to Day 730 post the Index Date

|  | **Cohort 1 (n = 143649)**  **Never received COVID-19 vaccine before the index date** | |  | **Cohort 2 (n = 29863)**  **Received COVID-19 vaccine before the index date** | |
| --- | --- | --- | --- | --- | --- |
|  | Patients with outcome (%) | HR (95% CI) |  | Patients with outcome (%) | HR (95% CI) |
| **Asthma or death** |  |  |  |  |  |
| Non-COVID-19 | 4010 (2.8%) | Reference |  | 1436 (4.8%) | Reference |
| COVID-19 | 8595 (6.0%) | 2.099 (2.022-2.179) |  | 3194 (10.7%) | 2.319 (2.179-2.468) |

1. Use COVID-19 Research Network.

|  | **Cohort 1 (n = 147272)**  **Never received COVID-19 vaccine before the index date** | |  | **Cohort 2 (n = 25586)**  **Received COVID-19 vaccine before the index date** | |
| --- | --- | --- | --- | --- | --- |
|  | Patients with outcome (%) | HR (95% CI) |  | Patients with outcome (%) | HR (95% CI) |
| **Asthma or death** |  |  |  |  |  |
| Non-COVID-19 | 2918 (2.0%) | Reference |  | 956 (3.7%) | Reference |
| COVID-19 | 6863 (4.7%) | 2.304 (2.206-2.406) |  | 2453 (9.6%) | 2.649 (2.459-2.855) |

Hazard ratio (HR) and 95% CI are provided, demonstrating outcomes among COVID-19 infected individuals versus non-infected counterparts

**Table S5: Codes for definitions of covariates, outcomes**

| **Variable** | **Code(s)** |
| --- | --- |
| SARS coronavirus 2 and related RNA | 9088 |
| Neoplasms | C00-D49 |
| Post COVID-19 Condition | Emergency use of U07 (U07)  Post COVID-19 condition (U09)  Personal history of COVID-19 (Z86.16) |
| COVID-19 Vaccines | mRNA-LNP vaccines (e.g., 91300, 91305, 91306, 91307, 91308, 91309, 91311, 91312, 91313, 91314, 91315, 91316, 91317, 91301).  Vector non-replicating vaccines (2479831).  Recombinant spike protein nanoparticle vaccines (91304).  Bivalent booster vaccines (230). |
| Asthma | Asthma (J45)  Other and unspecified asthma (J45.9)  Unspecified asthma (J45.90)  Unspecified asthma, uncomplicated (J45.909)  Other asthma (J45.99)  Mild intermittent asthma (J45.2)  Other asthma (J45.998)  Mild intermittent asthma, uncomplicated (J45.20)  Unspecified asthma with (acute) exacerbation (J45.901)  Moderate persistent asthma (J45.4)  Mild persistent asthma (J45.3)  Mild persistent asthma, uncomplicated (J45.30)  Moderate persistent asthma, uncomplicated (J45.40)  Mild intermittent asthma with (acute) exacerbation (J45.21)  Exercise induced bronchospasm (J45.990)  Moderate persistent asthma with (acute) exacerbation (J45.41)  Mild persistent asthma with (acute) exacerbation (J45.31)  Unspecified asthma with status asthmaticus (J45.902)  Severe persistent asthma (J45.5)  Severe persistent asthma, uncomplicated (J45.50) |
| Anti-Asthmatic Drug | Fluticasone (41126)  Adrenergics, Inhalants (R03A)  Leukotriene Receptor Antagonists (R03DC)  Antiasthmatic Other (RE109)  Corticosteroids for Systemic Use (H02)  Budesonide (19831)  Ciclesonide (274964)  Mometasone (108118)  Beclomethasone (1347)  Anti-Inflammatories, Inhalation (RE101)  Antiasmatic/Bronchodilators (RE100)  Bronchodilators, Sympathomimetic, Inhalation (RE102) |
| Severe COVID-19 was defined as having Hospital Inpatient Services, ICU, Mechanical ventilation records within 1 month after COVID-19 infection | Hospital Inpatient Services (1013659, 1013699, 1013729)  Critical Care Services (ICU) (1013729)  Mechanical Ventilation (31500, 1015098, 5A1935Z, 5A1945Z, 5A1955Z, 0BH17EZ, 0BH18EZ, 0BH13EZ, 1022227) |
