# Supplementary material for: The association between COVID-19 vaccine/infection and new-onset asthma in children - based on the global TriNetX database
Source: Infection. 2024 Jun 21;53(1):125–37. doi: 10.1007/s15010-024-02329-3 (PMC11825542; doi:10.1007/s15010-024-02329-3)
Supplement: Supplementary file 2 — Supplementary Material 2 [file 15010_2024_2329_MOESM2_ESM.docx]

**Supplementary**

**Figure S1: Kaplan-Meier curves of cumulative probability (%) of outcomes comparing COVID-19 and non-COVID-19 group, from Day 30 to Day 730 post the Index Date. Panels (a), (c), and (e) depict Cohort 1; Panels (b), (d), and (f) depict Cohort 2**

| 1. Asthma or death   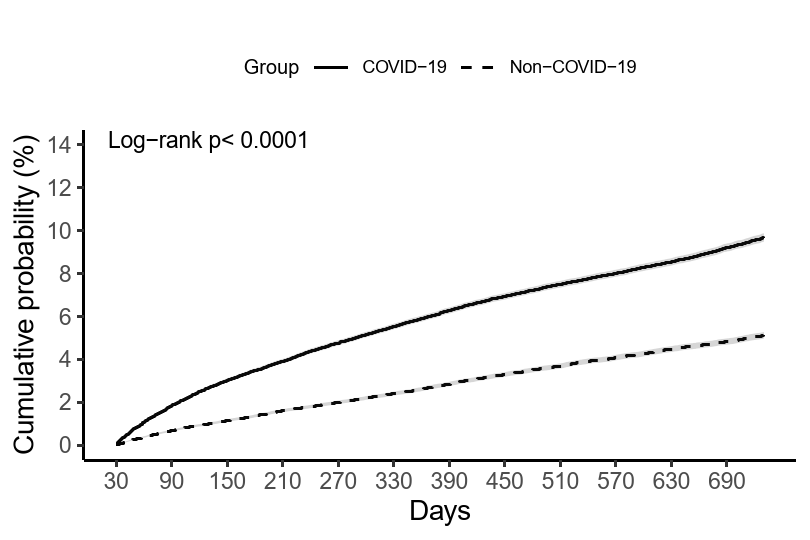 |  | 1. Asthma or death   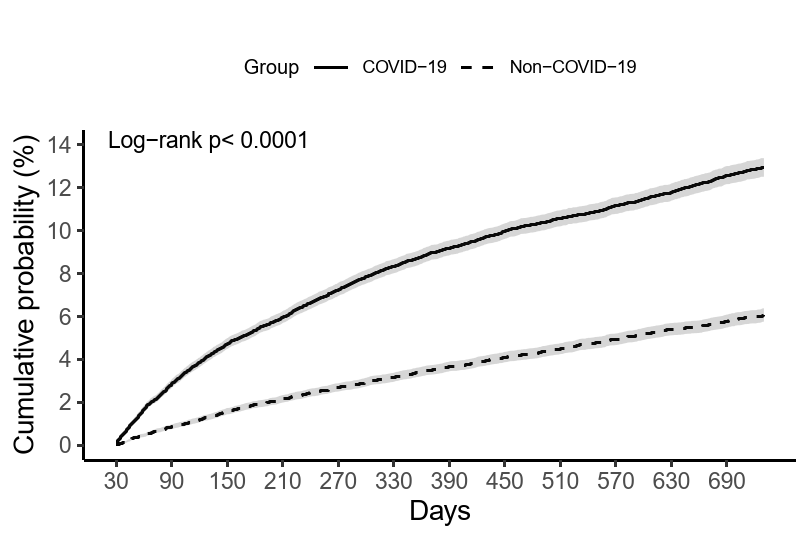 |
| --- | --- | --- |
| 1. Any anti-asthmatic drugs or death   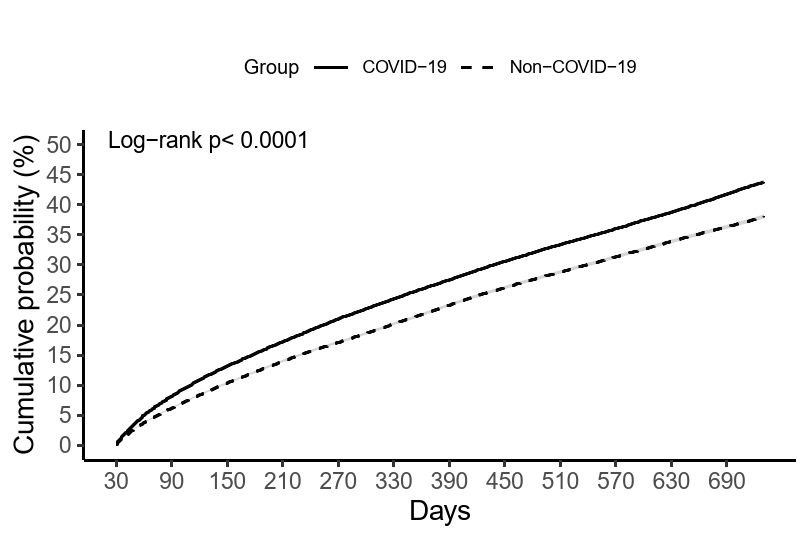 |  | 1. Any anti-asthmatic drugs or death   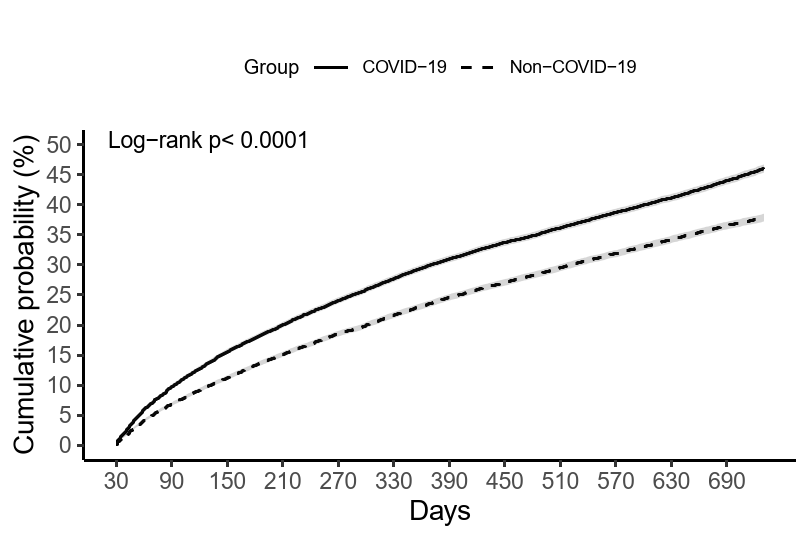 |
| 1. Asthma or anti-asthmatic drugs or death   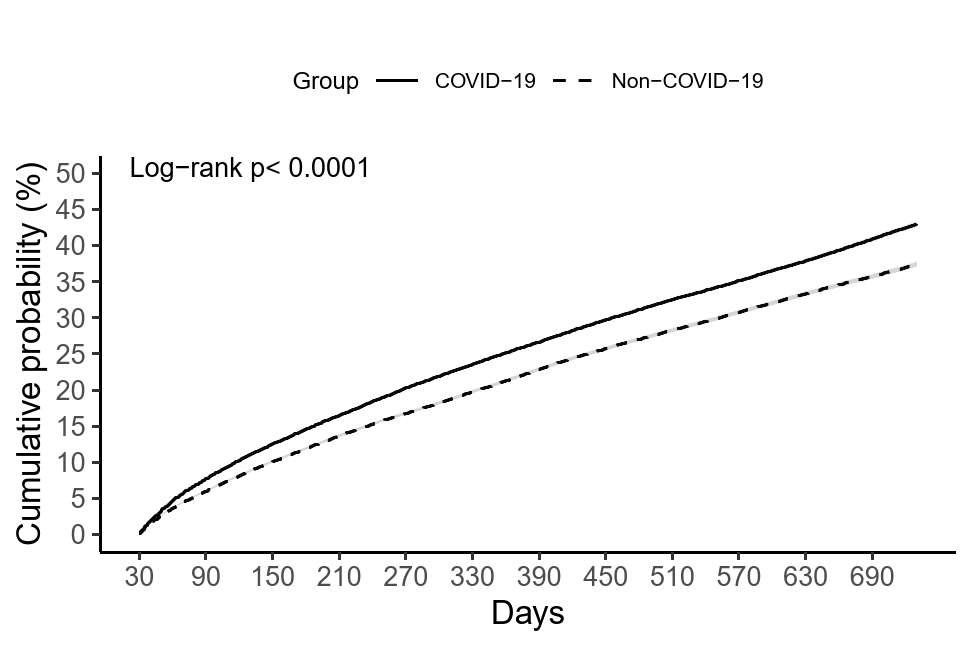 |  | 1. Asthma or anti-asthmatic drugs or death   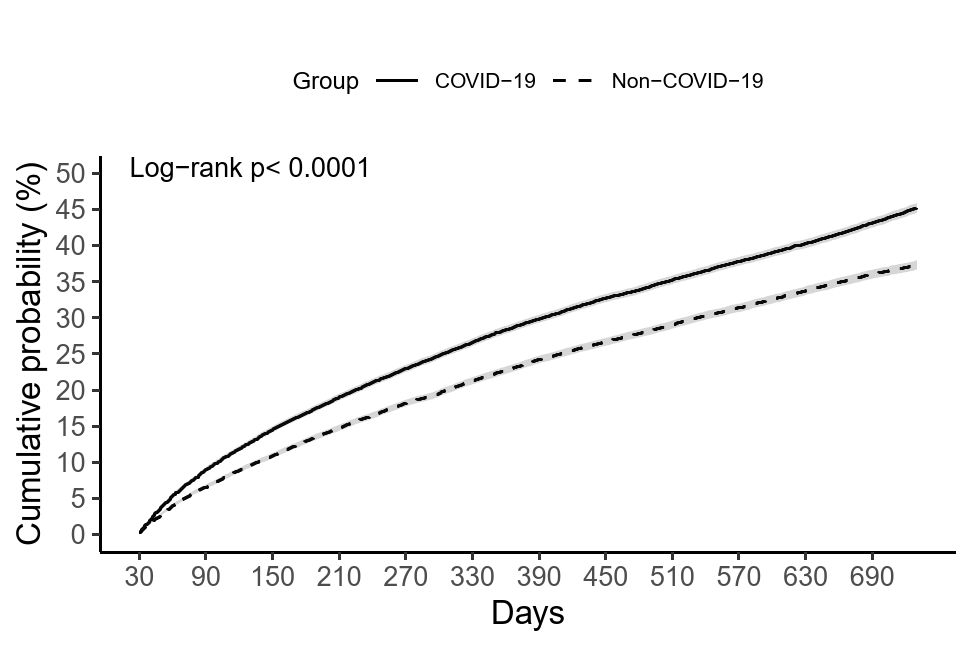 |
